# Conservative management of acute calculous cholecystitis complicated by pancreatitis in an elderly woman: A case report

**DOI:** 10.1097/MD.0000000000011200

**Published:** 2018-06-22

**Authors:** Marta K. Walczak-Galezewska, Damian Skrypnik, Monika Szulinska, Katarzyna Skrypnik, Pawel Bogdanski

**Affiliations:** a Department of Internal Diseases, Metabolic Disorders and Hypertension; b Department of Education and Obesity Treatment and Metabolic Disorders, Poznań University of Medical Sciences, ul. Szamarzewskiego; c Institute of Human Nutrition and Dietetics, Poznań University of Life Sciences, ul. Wojska Polskiego, Poznań, Poland.

**Keywords:** acute calculous cholecystitis, conservative management, pancreatitis, surgery

## Abstract

**Rationale:**

Acute calculous cholecystitis is a prevalent disease whose diagnosis and management still face significant debate. Although the overall incidence of gallstone disease is 18.8% in European women aged 30 to 69 years, there is little data and experience in managing acute calculous cholecystitis in populations over 80 years old. The incidence of acute cholecystitis among the elderly is probably increasing. For the reason, we here highlight the advantages and disadvantage of various treatment and management opens based on a 96-year-old patient.

**Patient concerns:**

We present a rare case in which a 96-year-old woman suffered from abdominal pain, nausea, and lack of appetite for over a month.

**Diagnoses:**

She was diagnosed with acute calculous cholecystitis and pancreatitis.

**Interventions:**

She was successfully treated without surgery, regaining her physical health after 5 months.

**Outcomes:**

The question of how to manage acute calculous cholecystitis is extremely difficult in many aspects. The patient of very advanced age presented in this paper, not very well diagnosed and with a life-threating condition, survived because of careful treatment and reasonable decision-making.

**Lessons:**

The take-away from this case is that, in a high-risk senile patient, strict conservative therapy of cholecystitis may be successful, as it can avoid the complications of surgery and leave the patient with a good quality of life.

## 1 Introduction

The prevalence of acute calculous cholecystitis (ACC) in the European female population aged 30 to 69 years is 18.8%, with biliary colic present in 1% to 4% a year.^[1]^ In Poland, cholecystolithiasis occurs in 30% of people older than 75 years and, within this group as many as 50% of patients are at increased risk of gallstone. The main reasons for this are aging processes that lead to an increase in cholesterol content in the bile, a decrease in gallbladder motility, a decrease in serum concentration of cholecystokinin, and a decreased cholecystokinin response.^[2]^ ACC, despite its well-known pathophysiology, still presents significant management challenges, particularly in patients aged 80 years and older.^[1,2]^ The 2007^[3]^ and 2013^[4]^ Tokyo guidelines (TG) provided recommendations for the diagnosis and treatment of ACC, but leave a broad margin of doubt, especially on the diagnostic value of single abdominal ultrasound (AUS) signs and the timing of surgery. Also, the needs of the operation (compared to conservative management), the definition and management options in high surgical risk patients, and the role of cholecystectomy in this group of patients have all been insufficiently discussed. Moreover, the proposed ACC scoring system has not been validated.^[1]^ Due to these shortcomings, the World Society of Emergency Surgery (WSES) convened a consensus conference and developed the most current guidelines on ACC diagnosis and treatment.^[1]^ In briefly, there is no single clinical or laboratory finding with sufficient diagnostic accuracy to establish or exclude ACC, so a combination of detailed history, complete clinical examination, and laboratory tests should be used. AUS is a gold-standard imaging diagnostic technique, while evidence for the accuracy of computed tomography (CT) and magnetic resonance imaging (MRI) in ACC diagnosis are scarce; hepatobiliary iminodiacetic acid scans show the greatest sensitivity and specificity for acute cholecystitis; however, these are not widely available. In the therapeutic range, it has been demonstrated that gallstone dissolution or extracorporeal shock wave lithotripsy (ESWL) plays no role in the treatment of ACC, and cholecystectomy represents the primary therapeutic option, superior to observational strategies. Antibiotics can provide supportive therapy in ACC, and are the primary therapy in patients undergoing observation or delayed surgery.^[1]^ It should be emphasized that, in the case of ACC, age above 80 years represents a significant risk factor for worse clinical behavior, morbidity, and mortality.^[1]^ On the other hand, in patients who have undergone conservative treatment of ACC, the probabilities of gallstone-related events (GRE) 6 weeks, twelve weeks, and one year after discharge from hospital are 14%, 19%, and 29%, respectively. The most frequent GRE are biliary colic (70%), biliary tract obstruction (24%), and pancreatitis (6%).^[5]^

The aim of this study is to present a case of acute calculous cholecystitis complicated by pancreatitis in a 96-year-old woman in the context of therapeutic doubts regarding surgical versus conservative management in elderly patients.

## 2 Case presentation

A 96-year-old woman was admitted to our hospital due to a 7-day history of fever over 39°C and 2 episodes of vomiting. By the time of admission, she had had abdominal pain, nausea, and lack of appetite for over a month. The family called an emergency ambulance but twice did not allow the patient to be taken to hospital. Because of her deteriorating condition, which was showing no improvement with oral amoxicillin with clavulanic acid 3 times a day (0.625 g), she was referred to our department to improve her poor condition. She had undergone appendectomy in her forties and hip bone fracture in 2013. She had not been administered any drugs so far.

Clinical examination revealed full mental conscious, dehydration, pulse rate of 90 beats/min, blood pressure 120/70 mm Hg, a body temperature of 39.5°C, left-side alignment of the alveolar murmur at the base of the left lung, local tenderness in the right upper abdomen without muscular defense. Her initial laboratory measurements showed a white blood cell (WBC) count of 17.77 × 10^9^/L (norm: 4 × 10^9^–10 × 10^9^/L), with 86.8% neutrophils, hemoglobin at 6.80 mmol/L (norm: 7.45–10.00 mmol/L), erythrocyte sedimentation rate (ESR) was 86 mm (norm: 3–15 mm), and C-reactive protein level was 177.00 mg/L (norm: 0.00–5.00 mg/L). AUS showed gallbladder enlargement with stones and widening of the common bile duct (CBD) to 16 mm. The patient was consulted by the surgeon who, due to the high risk of mortality, decided to carry out intravenous antimicrobial therapy with ciprofloxacin 2 times a day with a dose of 0.4 g, metronidazole 2 times a day with a dose of 0.5 g, intravenous rehydration and alimentation, and spasmolytic drugs. We observed a mild improvement in the patient's condition with a decrease of body temperature to 36.8°C. Unexpectedly, 10 days after admission, we observed aggravation of the abdominal pain and fever recurrence up to 39.9°C, with an increase in serum and urine amylase to 257 U/L (norm: 25–115 U/L) and 418 U/L (norm: 30–200 U/L), respectively. CT of the abdomen revealed gallbladder stones with a widening of the CBD to 13 mm and widening of the Virsung duct to 8 mm, as well as some small pancreatic cysts with a pancreas tumor of 38 mm in diameter. CT also revealed aneurysm of the abdominal aorta, left suprarenal tumor, and esophagus hernia that had not been previously diagnosed. Abdominal MRI confirmed calculous cholecystitis and pancreatitis, probably with inflammatory tumor of the pancreas. After multidisciplinary consultation, we decided to treat the patient with meropenem intravenously three times a day at a dose of 1.0 g. The patient did not consent to undergo surgery. Over the next few days, the patient's health significantly improved, and after three weeks of hospitalization she was discharged home, with neither fever nor abdominal pain. She received restricted dietary recommendations. After discharge, the patient further gradually regained her physical health and returned to the condition she had presented before the disease. Four months after hospitalization, her laboratory tests showed a WBC count of 6.09 × 109/L, with 72.5% neutrophils, hemoglobin at 7.70 mmol/L, ESR of 38 mm, and C-reactive protein level at 1.03 mg/L. The patient was administrated pancreatic enzymes twice a day at a dose of 10,000 units before meals. Her blood pressure was well-controlled and she has had no recurrence of the fever or abdominal pain.

## 3 Discussion

As stated by WSES, advanced age has been identified as a perioperative risk factor in ACC. Moreover, it has not been demonstrated that early laparoscopic cholecystectomy is the best treatment protocol in this group of patients. It has been reported that the mortality odds ratio in ACC patients aged over 80 years, even with low anesthetic risk, was higher than for groups aged 65 to 79 years and 50 to 64 years (30.9% vs 5.5% vs 1%).^[6]^ Also, there are significant differences in mortality (4.8% vs 0.5%), morbidity (31% vs 15%), and average hospital stay (3.9 vs 2.8 days) between patients suffering from ACC aged above and below 75 years respectively.^[7]^ Girgin et al^[8]^ have shown that patient's age, comorbidities, and Mannheim peritonitis index ≥ 29 are strongly connected with morbidity. Senility and low WBC count are also correlated with mortality in gangrenous cholecystitis.

On the other hand, a range of studies has shown that surgery should be the first-line treatment for symptomatic cholelithiasis and ACC in elderly patients.^[2,9]^ In the study of Arthur et al on 46 patients over 80 years of age with symptomatic cholelithiasis, conservative treatment was initially implemented in 23 cases. In 18 patients (nearly 80%), this was ineffective; it resulted in the patient's death in 4 cases (17%). In turn, of 23 patients who underwent open or laparoscopic surgery, only one death occurred, and only 6 patients presented complications that did not limit the good treatment effects^[2]^. In addition to this, elderly patients significantly more often develop immediate complications of cholelithiasis and ACC, such as acute pancreatitis, Mirizzi's syndrome, gastrointestinal fistula, and bile obstruction, in which surgery should be performed.^[9–12]^

Studies show that the mode of surgery is also crucial for ACC outcomes in elderly patients. The mortality of patients over 80 years of age is 34.2% in the case of urgent cholecystectomy, compared to 0% in the case of elective and semielective surgery. Morbidity and hospital stay duration are different in these groups. However, patients over 80 years who undergo urgent cholecystectomy, compared to those who endure elective or semielective operations, are usually at high anesthetic risk (American Society of Anesthesiologists score III and IV): 76% vs 25.6% vs 28.6%. A significantly lower number of laparoscopic cholecystectomies (20% vs 81.3% vs 82.8%) are performed in this particular group.^[13]^ On the other hand, a range of studies have shown no differences in mortality or postoperative complications when comparing early and delayed cholecystectomy in elderly patients with ACC.^[14–16]^

Taking into account the patient's lack of consent for surgery and its high risk, as well as the comorbidity (aneurysm of abdomen aorta, undiagnosed left suprarenal tumor), the patient's good clinical state, the low symptom intensity, and the expected good quality of life, we decided to use conservative management of ACC in the internal medicine ward. Due to the stable state of the patient, and in line with the WSES guidelines, ciprofloxacin and metronidazole were given, with conversion to meropenem after the occurrence of pancreatitis.^[1]^ Strict compliance with the therapy and appropriate dietary recommendations resulted in very good treatment effects. Considering that 38% of aged ACC patients who did not undergo surgery require readmission to hospital,^[17]^ the lack of clinical and laboratory symptoms of ACC in the patient at the four-month follow-up can be considered a treatment success. Patient comorbidities (aneurysm of abdominal aorta, left suprarenal tumor) introduce a need for further diagnosis and therapy.

## 4 Conclusion

Strict compliance with comprehensive guidelines on conservative management in a case of senile ACC patients with high surgery risk may result in a favorable treatment result, both during hospitalization and in follow-up, even despite the occurrence of pancreatitis.

## Author contributions

All authors were involved in the treatment of the patient described in the case report. MWG, DS, and KS wrote the first draft of the case report; MS, PB revised it critically for important intellectual content; all authors participated in the final revision of paper; all authors have read and have given final approval of the version to be published.

**Conceptualization:** Marta Walczak-Galezewska, Damian Skrypnik.

**Data curation:** Marta Walczak-Galezewska, Damian Skrypnik.

**Formal analysis:** Marta Walczak-Galezewska, Damian Skrypnik, Pawel Bogdanski.

**Funding acquisition:** Pawel Bogdanski, Monika Szulinska.

**Investigation:** Marta Walczak-Galezewska, Monika Szulinska.

**Methodology:** Marta Walczak-Galezewska, Monika Szulinska, Katarzyna Skrypnik, Pawel Bogdanski.

**Project administration:** Katarzyna Skrypnik.

**Software:** Katarzyna Skrypnik.

**Supervision:** Damian Skrypnik, Monika Szulinska, Pawel Bogdanski.

**Writing – original draft:** Marta Walczak-Galezewska, Damian Skrypnik, Katarzyna Skrypnik.

**Writing – review & editing:** Marta Walczak-Galezewska, Damian Skrypnik, Monika Szulinska, Katarzyna Skrypnik, Pawel Bogdanski.
